# Mislabeling Chronic Depression: A Case Report of Persistent Depressive Disorder Misdiagnosed for 12 Years

**DOI:** 10.7759/cureus.97835

**Published:** 2025-11-26

**Authors:** Tagwa Hegazi, Jasimah Jamir, Albra Hegazi, Yusra Elhassan

**Affiliations:** 1 Psychiatry, University Hospital Kerry, Tralee, IRL; 2 Urology, Sudan Medical Specialization Board, Khartoum, SDN; 3 Clinical Microbiology, Sudan Medical Specialization Board, Khartoum, SDN

**Keywords:** chronic depression, differential diagnosis, dysthymia, misdiagnosis, persistent depressive disorder

## Abstract

Persistent depressive disorder (PDD) is a chronic mood condition defined by a depressed mood lasting at least two years. However, it is frequently misdiagnosed as episodic depression, such as major depressive disorder (MDD) or recurrent depressive disorder (RDD), or attributed to personality-based pathology such as emotionally unstable personality disorder (EUPD). We present the case of a 48-year-old woman with a lifelong low mood and passive death wishes, who, for 12 years, received multiple shifting diagnoses and underwent extensive antidepressant and augmentation trials, as well as a full course of dialectical behaviour therapy (DBT), without achieving meaningful improvement. Longitudinal assessment demonstrated a continuous depressive baseline with intermittent past worsening, but no sustained remission exceeding two months, preserved occupational functioning, and absence of behavioural dysregulation, supporting a diagnosis of PDD with intermittent major depressive episodes, without current episode (Type 3). Misdiagnosis resulted from functional masking of severity, a clinical bias toward episodic interpretation of mood fluctuation, and misattribution of emotional sensitivity as personality pathology. Accurate recognition of PDD is clinically essential, as it reframes treatment goals toward long-term management rather than pursuit of full remission, stabilizes the therapeutic alliance, and prevents treatment fatigue and disengagement.

## Introduction

Persistent depressive disorder (PDD) is defined in the Diagnostic and Statistical Manual of Mental Disorders, Fifth Edition (DSM-5) as a mood disorder characterised by depressed mood present for most of the day, on more days than not, for at least two years [1]. The core feature is the chronicity of the low mood, a trait that differentiates it from the episodic mood disturbances seen in major depressive disorder (MDD). The importance of distinguishing PDD from other depressive disorders lies in this fundamental difference. While MDD is episodic, PDD represents a persistent mood state that often becomes part of the individual's emotional baseline [2].

Despite its clinical relevance, PDD remains frequently under-recognised or misdiagnosed [3]. Many individuals who present with longstanding depressive symptoms are instead labeled with MDD or recurrent depressive disorder (RDD) during periods of worsening symptoms, or with emotionally unstable personality disorder (EUPD) when chronicity and interpersonal sensitivity are misinterpreted as character pathology [4]. This diagnostic confusion is not merely academic; it carries significant therapeutic consequences, often resulting in fragmented care, inappropriate psychotherapeutic referral, and repeated pharmacological trial-and-error [5].

The present case report demonstrates that diagnostic accuracy in PDD is a therapeutic imperative. In this case, recognising PDD was the key intervention that disrupted over a decade of misdirected treatment and allowed the development of an effective, stable, and collaborative care plan.

## Case presentation

The case is of a 48-year-old single woman who presented with a lifelong, pervasive low mood, anhedonia, low self-esteem, and passive death wishes. Written informed consent for publication was obtained prior to preparing this case report. She maintains stable functioning, including sustained employment, independent living, and caregiving for her elderly parent. Despite this outward stability, she described a chronic depressive baseline dating back to adolescence.

She reported feeling low “most days, almost all the time” with a persistent loss of enjoyment in activities, stating, “Life looks grey. I don’t remember ever being happy.” She also described passive death wishes, such as “I’m alive because I’m not dead,” but denied suicidal intent or planning, noting that her religious beliefs prevented her from considering self-harm. In addition, she expressed longstanding low self-esteem, reporting chronic feelings of inadequacy regarding her appearance and life achievements.

She denied neurovegetative symptoms typical of episodic major depression, including changes in appetite, sleep disturbance, psychomotor changes, impaired concentration, or decisional difficulties.

Her psychosocial history reflected a remarkably stable and coherent life trajectory. She completed higher education in interior design, maintained steady employment throughout adulthood, and reported positive, supportive relationships with her three siblings. She has always lived independently, manages her finances without difficulty, does not use substances, and denies any legal, interpersonal, or occupational instability. She reported no childhood trauma and described her upbringing as emotionally safe and structured.

Her presentation lacked features commonly associated with personality pathology. She had no history of self-harm, impulsivity, interpersonal instability, suicidal behaviour, or crisis-driven service use. Her distress was characterised by quiet endurance and emotional resignation rather than behavioural dysregulation.

She reported no family history of mental illness. Her medical history was notable only for a lumbar disc prolapse, and she denied use of alcohol, tobacco, or illicit substances.

Routine investigations were completed during assessment, including full blood count, thyroid function tests, metabolic profile, and B12/folate levels, all of which were within normal limits. There was no clinical or biochemical evidence of a medical or neurological condition contributing to her symptoms. Collateral history was unavailable from family members, although the patient reported stable, supportive relationships, and relatives raised no concerns throughout her care.

Diagnostic and treatment history

The patient's 12-year contact with mental health services was characterised by shifting diagnostic labels that reflected clinicians’ focus on presenting symptom fluctuations rather than the underlying chronic depressive baseline. She was initially diagnosed with MDD in 2012. Between 2014 and 2017, this was revised to RDD with comorbid generalised anxiety disorder (GAD), as periods of increased distress were interpreted as discrete depressive episodes. In 2017-2018, her presentation was reframed within a personality-based formulation, and she was diagnosed with EUPD due to descriptions of emotional sensitivity. From 2020 to 2023, her records reflected a hybrid formulation of MDD with GAD and “EUPD traits,” indicating ongoing diagnostic uncertainty. In 2024, the diagnosis was once again recorded as RDD.

This diagnostic variability led to extensive pharmacological interventions, including multiple selective serotonin reuptake inhibitor (SSRI) and serotonin-noradrenaline reuptake inhibitor (SNRI) trials (sertraline, fluoxetine, escitalopram, venlafaxine), a tetracyclic antidepressant (dothiepin), and augmentation with quetiapine, olanzapine, and sodium valproate. Despite these medication adjustments, she reported minimal sustained improvement. Based on the EUPD formulation, she completed a full course of dialectical behaviour therapy (DBT), which she found helpful for understanding emotional responses but ineffective in altering her chronic baseline mood state. 

A summary of the diagnostic changes and treatment trials across the 12-year period is provided in Table 1.

**Table 1:** Summary of diagnoses and treatments (2012-2024) MDD, major depressive disorder; SSRI, selective serotonin reuptake inhibitor; DBT, dialectical behaviour therapy; GAD, generalised anxiety disorder; EUPD, emotionally unstable personality disorder; ECT, electroconvulsive therapy; RDD, recurrent depressive disorder

Year(s)	Diagnosis	Treatment(s)
2012	MDD	SSRI trial (sertraline)
2014-2017	RDD + GAD	Fluoxetine, escitalopram, venlafaxine
2017-2018	EUPD	DBT started after diagnosis, sodium valproate, olanzapine
2020-2023	MDD + GAD + "EUPD traits"	Dothiepin; augmentation with quetiapine
2024	RDD	Ongoing medication adjustments; request for ketamine/ECT

Repeated re-diagnoses and treatment trials contributed to treatment fatigue and episodic disengagement from services. She described her care as “changing medications without changing anything about how I feel,” which led her to request escalation to higher-intensity interventions such as ketamine therapy or electroconvulsive therapy. Overall, her clinical trajectory reflects chronic depressive symptoms misinterpreted as episodic or personality-based, resulting in fragmented and ultimately ineffective treatment [5]. No structured follow-up occurred, as the patient subsequently disengaged from services consistent with her long-standing pattern of treatment fatigue in the context of chronic depressive illness.

## Discussion

This discussion examines over a decade of misdiagnosis, the factors concealing the patient’s true condition, and an evidence-based rationale for revising the diagnosis to PDD, which explains her lifelong low mood and matches the chronic depressive course found in contemporary literature [6].

Diagnostic challenges

The patient’s history reveals a series of reasoning mistakes, with four main factors contributing to the diagnostic error. First, functional masking: her ability to maintain employment and live independently caused clinicians to overestimate her well-being. This is a common reason chronic depression is overlooked despite significant internal impairment [6,7].

A second factor was episodic bias. Fluctuations in her distress were repeatedly interpreted as discrete episodes, resulting in diagnoses of MDD or RDD. The failure to consider longitudinal course rather than cross-sectional severity remains a major source of diagnostic inaccuracy in chronic depression [2,6].

A third factor involved the misattribution of emotional sensitivity and a persistently negative self-concept to EUPD, despite the absence of hallmark features such as behavioural dysregulation, identity disturbance, or interpersonal instability [4]. Previous literature has highlighted that chronic depressive traits are frequently mistaken for personality pathology, particularly in women [6].

The final factor was therapeutic misalignment. The patient was referred for DBT, a treatment intended for personality-related emotional dysregulation, rather than interventions designed for chronic depressive mood. Repeated failures of medication adjustments and inappropriate psychotherapy reinforced the patient’s perception that clinicians were “changing the name, not the problem,” contributing to treatment fatigue and disengagement [5-8].

Evidence from recent literature on chronic depression

PDD frequently presents with subtle yet longstanding symptoms and normal outward functioning, which can delay recognition for years. Contemporary reviews emphasise that chronic depression may appear “mild” when viewed cross-sectionally but is significantly disabling when assessed longitudinally [6]. This aligns with the clinical trajectory observed in this case.

Recent work has highlighted the need for earlier and more systematic detection of PDD. Upadhyay et al. used questionnaires based on the Diagnostic and Statistical Manual of Mental Disorders, Fourth Edition (DSM-4)/DSM-5 criteria and standard rating scales, such as the Hamilton and Cornell Dysthymia Rating Scales, to train a stacked support vector machine model for early PDD screening, achieving nearly 90% accuracy [9]. Although conducted in a non-clinical undergraduate population, these findings reinforce that chronic depressive patterns are detectable when systematically assessed. The absence of structured longitudinal assessment and scale-based monitoring contributed directly to more than a decade of diagnostic drift in this case.

Treatment implications and alignment with guidelines

Clinical guidelines highlight the need for long-term management of chronic depression. National Institute for Health and Care Excellence (NICE) recommends using psychotherapies tailored for chronic depression, such as cognitive behavioral analysis system of psychotherapy (CBASP), interpersonal psychotherapy (IPT), cognitive behavioural therapy (CBT), or mindfulness-based cognitive therapy, along with steady and consistent medication management [10].

Pharmacotherapy remains an important component of treatment for PDD. Research shows that different types of antidepressants, like SSRIs, SNRIs, tricyclic antidepressants (TCAs), and monoamine oxidase inhibitors (MAOIs), are effective in chronic depression, with SSRIs generally better tolerated [11]. More noradrenergic agents such as mirtazapine, bupropion, venlafaxine, and duloxetine may also be beneficial in individuals with long-standing depressive symptoms [11]. Using both antidepressants and structured psychotherapy together has been shown to outperform either modality alone in dysthymia and chronic depression [11].

CBASP is especially effective for chronic depression and works better than medication by itself [12]. This is why DBT did not help this patient, since DBT is meant for emotional issues in personality disorders, not for chronic depression. Changing medications often, as happened here, is not recommended for chronic depression. These changes can create false hope for quick improvement without dealing with the ongoing nature of the illness [10]. A steady, diagnosis-based approach can help prevent treatment fatigue and support long-term engagement.

Clinical lessons from the case

This case highlights why it is important to assess patients over time, evaluate how their symptoms affect daily life, and use therapy tailored to the disorder when identifying PDD. PDD can show up in people who seem to function well but are struggling inside. Recognizing it early helps prevent misdiagnosis, reduces harm, and encourages steady, team-based care. In this case, however, there was no structured follow-up as the patient disengaged shortly after the revised diagnosis, expressing frustration at yet another diagnostic change after 12 years of inconsistent formulations and treatment approaches.

## Conclusions

This case shows how important it is to tell PDD apart from episodic mood and personality disorders. For 12 years, misdiagnosis led to fragmented care, many ineffective medication trials, and therapy that did not fit, which left the patient tired of treatment and disengaged. The confusion happened because clinicians focused only on acute symptoms and missed the long-term, ongoing depression that was hidden by her ability to function outwardly.

The primary clinical lesson from this case is that diagnostic precision is fundamental to effective therapeutic intervention. Correctly identifying PDD reframes the entire clinical approach, shifting goals from cure to management, prioritizing therapeutic alliance, and enabling the use of targeted treatments, such as CBASP. It requires clinicians to adopt a longitudinal perspective and develop the awareness needed to identify the grey, not only the acute depressive black, in chronic mood presentations.
